# Auranofin-Mediated NRF2 Induction Attenuates Interleukin 1 Beta Expression in Alveolar Macrophages

**DOI:** 10.3390/antiox10050632

**Published:** 2021-04-21

**Authors:** Stephanie B. Wall, Rui Li, Brittany Butler, Ashley R. Burg, Hubert M. Tse, Jennifer L. Larson-Casey, A. Brent Carter, Clyde J. Wright, Lynette K. Rogers, Trent E. Tipple

**Affiliations:** 1 Neonatal Redox Biology Laboratory, University of Alabama at Birmingham, Birmingham, AL 35294, USA; smoore@peds.uab.edu (S.B.W.); liri@uab.edu (R.L.); 2 Division of Neonatology, University of Alabama at Birmingham, Birmingham, AL 35294, USA; 3 Section of Neonatology, University of Colorado School of Medicine and Children’s Hospital Colorado, Aurora, CO 80045, USA; Brittany.Butler@childrenscolorado.org (B.B.); Clyde.Wright@UCDenver.edu (C.J.W.); 4 Department of Microbiology, University of Alabama at Birmingham, Birmingham, AL 35294, USA; Burga@uab.edu (A.R.B.); Htse@uab.edu (H.M.T.); 5 Pulmonary, Allergy, and Critical Care Medicine, University of Alabama at Birmingham, Birmingham, AL 35294, USA; jlcasey1@uab.edu (J.L.L.-C.); bcarter1@uab.edu (A.B.C.); 6 Birmingham Veterans Affairs Medical Center, Birmingham, AL 35233, USA; 7 Department of Pediatrics, University of Colorado School of Medicine and Children’s Hospital Colorado, Aurora, CO 80045, USA; 8 Center for Perinatal Research, Abigail Wexner Research Institute at Nationwide Children’s Hospital, Columbus, OH 43215, USA; Lynette.Rogers@Nationwidechildrens.org; 9 Section of Neonatal-Perinatal Medicine, University of Oklahoma Health Sciences Center, Oklahoma City, OK 73104, USA

**Keywords:** hyperoxia, auranofin, NFκB, NRF2, Il-1β

## Abstract

Background: Alveolar macrophages (AMs) are resident inflammatory cells in the lung that serve as early sentinels of infection or injury. We have identified thioredoxin reductase 1 inhibition by gold compounds increases activation of nuclear factor erythroid 2-related factor 2 (NRF2)-dependent pathways to attenuate inflammatory responses. The present studies utilized murine alveolar macrophages (MH-S) to test the hypothesis that the gold compound, auranofin (AFN), decreases interleukin (IL)-1β expression through NRF2-mediated interactions with nuclear factor kappa-light-chain-enhancer of activated B cells (NF-κB) pathway genes and/or increases in glutathione synthesis. Methods: MH-S cells were treated with AFN and lipopolysaccharide (LPS) and analyzed at 6 and 24 h. The *Il1b* promoter was analyzed by chromatin immunoprecipitation for direct interaction with NRF2. Results: Expression of IL-1β, p-IκBα, p-p65 NF-kB, and NOD-, LRR-, and pyrin domain-containing protein 3 were elevated by LPS exposure, but only IL-1β expression was suppressed by AFN treatment. Both AFN and LPS treatments increased cellular glutathione levels, but attenuation of glutathione synthesis by buthionine sulfoximine (BSO) did not alter expression of Il-1β. Analysis revealed direct NRF2 binding to the *Il1b* promoter which was enhanced by AFN and inhibited the transcriptional activity of DNA polymerase II. Conclusions: Our data demonstrate that AFN-induced NRF2 activation directly suppresses IL-1β synthesis independent of NFκB and glutathione-mediated antioxidant mechanisms. NRF2 binding to the promoter region of *IL1β* directly inhibits transcription of the *IL1β* gene. Collectively, our research suggests that gold compounds elicit NRF2-dependent pulmonary protection by suppressing macrophage-mediated inflammation.

## 1. Introduction

Acute respiratory distress syndrome (ARDS), a serious health disorder affecting both children and adults, involves the activation of inflammatory signaling resulting in lung injury. Inflammatory responses are driven, in part, by alveolar macrophages (AMs), resident inflammatory cells in the lung that serve as early sentinels of infection or injury [1]. As early mediators, macrophages become activated by stimuli and produce cytokines that subsequently propagate signaling and recruitment of other inflammatory cells. Uncontrolled inflammation driven by dysregulated macrophage signaling can contribute to chronic injury and disease progression. Therapeutic interventions often target inflammatory responses.

Previous work by our group identified thioredoxin reductase (Txnrd) 1 inhibition by gold compounds as a means to increase activation of nuclear factor erythroid 2-related factor 2 (NRF2)-dependent pathways to attenuate lung injury [2]. FDA-approved pharmaceutical gold compounds, such as aurothioglucose (ATG) and auranofin (AFN), decrease inflammation in the context of hyperoxic exposure and many other inflammatory diseases [3]. We and others have described a reduction in inflammatory cell numbers and cytokine expression in the lung following treatment with ATG or AFN [4,5,6,7]. Isakov et al. reported that macrophages treated with AFN and subsequently induced by lipopolysaccharide (LPS) demonstrated selective repression of inflammatory genes such as interleukin (IL)-1 [4].

The nuclear factor kappa-light-chain-enhancer of activated B cells (NFκB) family of transcription factors are key mediators of inflammation and, as such, are targets for therapeutic interventions designed to halt lung injury progression [8,9]. NFκB activation is regulated primarily through posttranslational mechanisms. Specifically, phosphorylation and proteolysis of the inhibitory proteins IκBα, IκBβ, and IκBε enables interaction of other NFκB subunits (p50, p65 NFκB) with promoter regions of target genes that regulate pro-inflammatory pathway activation. All cytosolic IκB isoforms function to prevent NFκB nuclear translocation and activation. However, when activation occurs, nuclear IκBβ has the unique role of enhancing NFκB subunit binding and stabilization at promoter regions of target genes [10]. Complete inhibition of NFκB activity has proven detrimental, indicating an essential function for homeostatic expression of this molecule. IL-1 as well as the components of the NOD-, LRR-, and pyrin domain-containing protein 3 (NLRP3) inflammasome are regulated by NFκB and respond to bacterial infection and oxidative stimuli. Some have postulated that suppression of IL-1 cytokines are mediated by NLRP3; however, the exact mechanism has not been defined.

There has been prolonged interest in the interaction(s) between the NFκB and NRF2 pathways [11]. Several studies have proposed that NRF2 activation increases antioxidant enzymes and, therefore, decreases oxidative burden by enhancing glutathione synthesis to relieve the oxidative effects of inflammation. Recent reports have identified a more direct role for NRF2 in the regulation of NFκB-dependent pathway activation [5,6,12]. Isakov et al. indicated the effects of Txnrd1 inhibition on IL-1β activation were likely to be upstream of glutathione (GSH) synthesis and related to transcriptional regulation [4].

The present studies utilized murine alveolar macrophages (MH-S) to examine the mechanisms by which AFN decreases pro-inflammatory IL-1β expression through NRF2-mediated interactions with NFκB pathway genes and/or increases in glutathione synthesis.

## 2. Materials and Methods

### 2.1. Cell Culture

Murine alveolar macrophages (MH-S; ATCC, CRL-2019) were maintained in RPMI media supplemented with 10% FBS, 8 mM glutamine, and penicillin/streptomycin (10,000 U). Cells were plated at equal densities. Our previous studies reported that 0.5 µM AFN (A6733, Sigma Aldrich, St. Louis, MO, USA) decreased Txnrd1 activity to less than 10% in the absence of toxicity 0 [2]. Thus, a concentration of 0.5 μM AFN was used for all further studies.

**Figure 1:**
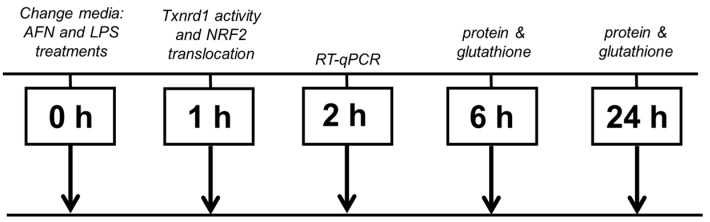
The time course for analyses. MH-S cells were cultured, exposed to lipopolysaccharide (LPS), buthionine sulfoximine (BSO), and/or auranofin (AFN), and harvested at the time points that were relevant for the respective endpoint. Phosphorylation of cytoplasmic factors, IκBα and p65 NFκB, and nuclear localization of NRF2 and NFκB occurred rapidly and were assessed at 1 h, and for transcripts of NLRP3 and IL-1β a timepoint of 2 h was chosen. Both protein and glutathione levels were measured at either 6 or 24 h to allow enough time for transcription and translation of IL-1β and other protein mediators of de novo glutathione synthesis.

At ~80–90% confluence, cells were treated in freshly changed full serum media with 0.5 μM AFN or vehicle control dimethyl sulfoxide (DMSO, BP-231, ThermoFisher, Waltham, MA, USA) and/or 0.5 µg/mL LPS. For experiments blocking de novo glutathione synthesis, buthionine sulfoximine (B2515, BSO, Sigma Aldrich, St. Louis, MO, USA) was used at a final concentration of 22.5 µM. Cells were harvested at time points optimal for each specific analysis; nuclear localization occurred rapidly and was observed at 1 h, changes in mRNA transcription were observed at 2 h, and protein translational increases were observed at 6 or 24 h. At the indicated times for each analysis (see Figure 1), cells were washed with Dulbecco’s phosphate-buffered saline and scraped in lysis buffer, 10 mM Tris buffer, pH 7.4, containing 0.1% Triton-X-100, 100 µM diethylenetriamine pentaacetic acid, and protease and phosphatase inhibitors (A32963, ThermoFisher, Waltham, MA, USA). Supernatant (obtained by centrifugation at 16,000× *g* × 10 min) protein concentrations were determined by bicinchoninic acid (BCA) assay (#23223, Thermo Scientific (Pierce), Waltham, MA, USA).

### 2.2. IL-1β ELISA

Cells were harvested 6 h after treatment with AFN/LPS, and ELISAs were performed on 20× dilutions of each sample using the manufacturer’s protocol for the DuoSet ELISA for Mouse IL-1β (DY201, R&D Systems, Minneapolis, MN, USA). Data were log-transformed for linear regression analysis, and values were normalized by the respective protein concentrations measured via BCA assay.

### 2.3. Nuclear Fractionation

Confluent cells in 6-well plates were separated for cytoplasmic or nuclear fractions similar to Michaelson et al. [13]. Cells were collected into 100 µL of 10 mM Tris, pH 7.4, 10 mM NaCl, 3 mM MgCl_2_, and protease inhibitors and centrifuged at 3000× *g* for 1 min. The cell pellet was lysed with 20 µL of the same buffer with the addition of 10% glycerol, 0.25% NP-40, and 1 mM DTT and centrifuged at 10,000× *g* for 1 min. The supernatant, or the cytoplasmic fraction, was collected into a fresh tube. The pellet was rinsed with the cytoplasmic lysis solution and once with lysis buffer containing only 10% glycerol. The final nuclear pellet was resuspended in 20 µL of the cytoplasmic lysis solution, and proteins were separated as described below.

### 2.4. Immunoblot

Samples were heated at 95 °C for 5 min in the presence of 1× Laemmli buffer. For phosphorylation blots, whole-cell lysates were equally loaded by volume into gels. For nuclear fractions the samples were loaded by equal volume. Samples were loaded onto 4–15% Criterion™ or Mini-PROTEAN^®^ TGX™ gels (Bio-Rad) and proteins separated, transferred to PVDF or nitrocellulose membranes (Trans-Blot^®^, Bio-Rad), blocked with 5% milk in Tris-buffered saline containing 0.05% Tween-20, and probed with anti-phospho-NF-κB-p65 (ser 536)(#3033), anti-NFκB-p65 (#3034), anti-phospho-IκB-α (S32/S36) (#9246), anti-IκB-α (#9242) (Cell Signaling Technology, Danvers, MA, USA), or anti-NRF2 antibody (generated in collaboration with Dr. Edward Schmidt), followed by goat anti-rabbit IgG-HRP secondary antibody (Santa Cruz Biotechnology; 1:5000). Membranes were developed using Clarity^TM^ ECL Substrate (#1705060, Bio-Rad, Hercules, CA, USA) and imaged using a ChemiDoc^TM^ System (Bio-Rad). Phosphorylation blots were visualized on an Odyssey CLx Imager with Image Studio v4.0 software using secondary anti-rabbit (#926-68071) or anti-mouse Abs (#929-80020) (1:20,000 in 5% milk TBST) antibodies conjugated to either IRDye 680RD or IRDye 800CW (LICOR, Lincoln, NE, USA). For loading control, membranes were reprobed with either anti-GAPDH (ABS16, Millipore, Burlington, MA, USA), anti-β-actin (sc-1615, Santa Cruz, Dallas, TX, USA or A1978, Sigma Aldrich, St. Louis, MO, USA), or anti-nucleolin antibody (ab22758, Abcam, Cambridge, UK).

### 2.5. Quantitative Real-Time RT-PCR

Cells were harvested 2 h after treatment with AFN/LPS, and quantitative polymerase chain reaction (RT-PCR) was performed with purified RNA (RNaeasy kit; Qiagen). Approximately 0.1 µg of RNA was reverse transcribed into cDNA using a high-capacity cDNA reverse transcription kit (#4368813, ThermoFisher (Invitrogen), Waltham, MA, USA) under recommended thermal cycling settings (SimpliAmp; Life Technologies, , Carlsbad, CA, USA). Ten nanograms of cDNA was applied for PCR reactions performed using SsoAdvance Universal SYBR green supermix (#1725270, Bio-Rad, Hercules, CA, USA) and, according to manufacturer instructions, using a Bio-Rad iQ5 system. Quantification of mRNA was calculated by the comparative CT method and was presented as fold change of expression (2^−∆∆CT^) normalized to *GAPDH* mRNA levels. Murine *nlrp3*, *IL1b*, and *gapdh* were amplified using the following primer sets: *nlrp3* (forward: 5′-CACGTGGTTTCCTCCTTTTG-3′ and reverse: 5′-TCCGGTTGGTGCTTAGACTT-3′), *IL1b* (forward: 5′-AGAGCTTCAGGCAGGCAGTAT-3′ and reverse: 5′-GAAGGTGCTCATGTCCTCATC-3′), and *gapdh* (forward: 5′-AGGTTGTCTCCTGCGACTTC-3′ and reverse: 5′-ACTCCTTGGAGGCCATGTAG-3′).

### 2.6. Glutathione Recycling Assay

Cells were harvested 6 or 24 h after treatment with AFN/LPS, and total glutathione (GSH + 2GSSG) levels were assessed in cellular lysates via the Tietze recycling assay [14]. Oxidized glutathione (GSSG) was measured by reacting samples with 2-vinylpyridine for 1 h [15].

### 2.7. Chromatin Immunoprecipitation (ChIP)

Chromatin was isolated from cell pellets using the Magna ChIP G kit (17-611, Millipore, Burlington, MA) per protocol (Millipore). Sonication was performed using the Diagenode Bioruptor Plus for two sets of five 30 s cycles. DNA was quantified using a spectrophotometer, and 50 μg of chromatin was diluted to a total volume of 500 μL in dilution buffer. Antibodies used for immunoprecipitation included rabbit IgG (Millipore), anti-NRF2 (#12721, Cell Signaling Technology, Danvers, MA, USA), and anti-POL2 (05-952-I, Millipore, Burlington, MA, USA). Immunoprecipitations were incubated at 4 °C overnight. Enrichment of the IL-1β promoter was assessed by RT-PCR using SYBR green reagent (Qiagen) primers targeting the IL-1β promoter known to bind DNA polymerase II (POL2) (Fwd AGATGCTCTGGAAGGAAGCA; Rev GGCAGCTCCTGTCTTGTAGG) and NRF2 (F TGATGATGTTGGCAAAGGAA; R AAAAGCTAGAGTGCCCGTCA) [12]. Results were expressed as fold enrichment over IgG.

### 2.8. Statistics

Biochemical analyses (mean ± SEM) were analyzed using Graph Pad Prism^®^ 6.0 (GraphPad Software, San Diego, CA, USA) by unpaired *t*-test or 2-way ANOVA followed by Tukey’s multiple comparison tests. For ChIP comparisons between groups, the null hypothesis that no difference existed between treatment means was tested by the Mann–Whitney test. Statistical significance was accepted at *p* < 0.05.

## 3. Results

### 3.1. Effects of LPS and/or AFN on IL-1β Expression

**Figure 2:**
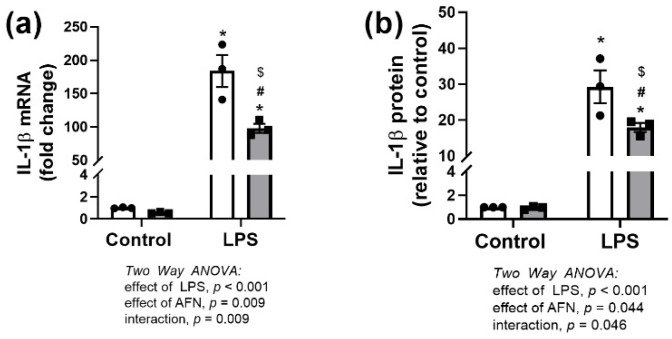
IL-1β is suppressed by AFN. (**a**) IL-1β mRNA (collected at 2 h) and (**b**) protein (collected at 6 h) was measured in LPS and AFN-treated MH-S cells. White bars indicate DMSO control, and grey bars indicate AFN-treated. Data were analyzed by two-way ANOVA to identify the effects of LPS and/or AFN exposure. Tukey’s post-hoc analysis was used to determine significant differences between groups; *n* = 3 individual experiments. * *p* < 0.005 from control non-stimulated; ^#^ *p* < 0.005 from AFN non-stimulated; ^$^ *p* < 0.05 from control LPS.

MH-S cells were treated with LPS and/or AFN. We detected independent effects of, and an interaction between, LPS and AFN on IL-1β mRNA and protein expression (Figure 2a,b). LPS-induced *Il1β* expression was decreased by approximately 50% in the presence of AFN (Figure 2a). Similarly, AFN attenuated IL-1β protein expression induced by LPS exposure (Figure 2b). 

### 3.2. NFκB Pathway Responses to LPS and/or AFN

**Figure 3:**
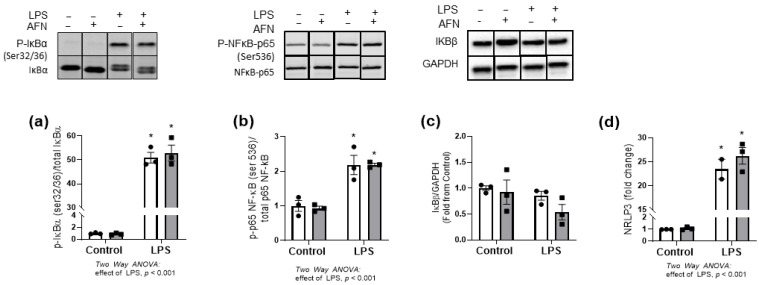
NFκB pathway is not affected by AFN. Protein levels of (**a**) p-IκBα, (**b**) p-p65 NFκB, and (**c**) total IkBβ were measured by immunoblot in LPS and AFN-treated MH-S cells at 1 h after treatment. (**d**) mRNA expression of NRLP3 was measured by RT-PCR 2 h after treatment and expressed as fold change. White bars indicate DMSO control, and grey bars indicate AFN-treated. Data were analyzed by two-way ANOVA to identify the effects of LPS and/or AFN exposure. Tukey’s post-hoc analysis was used to determine significant differences between groups; *n* = 3 individual experiments. * *p* < 0.005 from control non-stimulated.

To evaluate the effects of LPS and AFN on NF-κB activation, we assessed the phosphorylation of nuclear factor of kappa light polypeptide gene enhancer in B-cells inhibitor (IκB)α and the NF-κB subunit p65 in whole lysates, as well as cytosolic IκBβ degradation and expression of the NF-κB regulated gene, *Nrlp3*. As expected, we identified an independent effect of LPS on the phosphorylation of IκBα (ser32/36), NFκB-p65 (ser 536), and *Nrlp3* expression, with no change in IκBβ protein levels (Figure 3a–d and Supplementary Material Figure 3a–c). However, our data did not indicate an effect of AFN on pIκBα, p65 NF-kB, IκBβ, or *Nrlp3*. 

### 3.3. LPS and AFN Modulate Glutathione Levels

**Figure 4:**
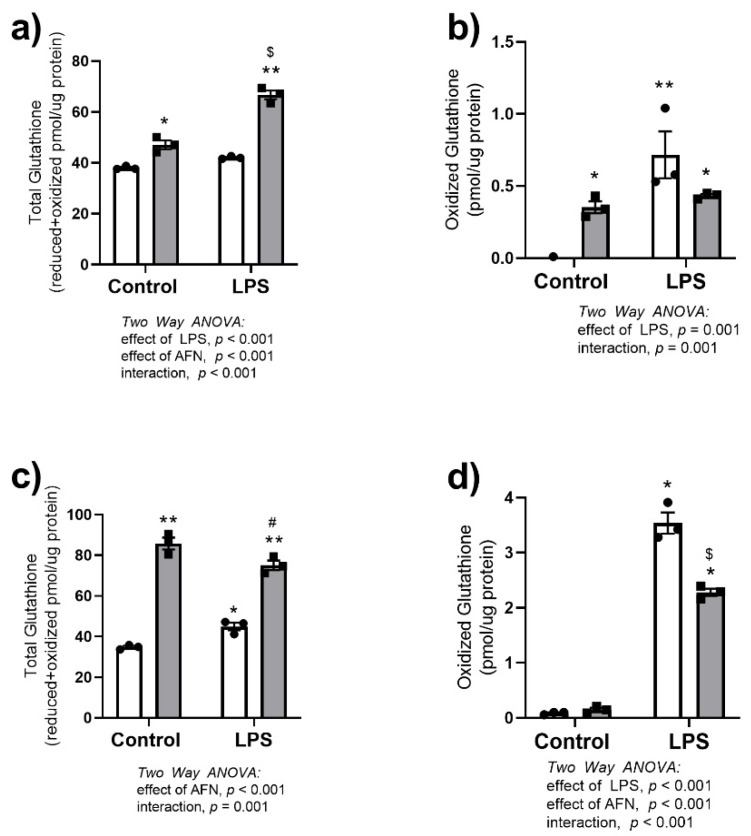
Total and oxidized glutathione are increased in response to AFN treatment. Cellular concentrations of (**a**) total glutathione (reduced + oxidized) and (**b**) oxidized glutathione were measured at 6 h, and (**c**) total glutathione (reduced + oxidized) and (**d**) oxidized glutathione were measured at 24 h in LPS and AFN-treated MH-S cells. White bars indicate DMSO control, and grey bars indicate AFN-treated. Data were analyzed by two-way ANOVA to identify the effects of LPS and/or AFN exposure. Tukey’s post-hoc analysis was used to determine significant differences between groups; *n* = 3 individual experiments. * *p* < 0.005 from control non-stimulated; ** *p* < 0.001 from control non-stimulated; ^#^ *p* <0.005 from AFN non-stimulated; ^$^ *p* < 0.05 from control LPS.

The effects of LPS and AFN on glutathione synthesis were evaluated. Two-way ANOVA indicated independent effects of an interaction between LPS and AFN on total glutathione at 6 h and GSSG at 24 h (Figure 4a,d). Specifically, LPS + AFN synergistically enhanced total glutathione levels at 6 h (Figure 4a) while attenuating GSSG levels at 24 h (Figure 4d). Total glutathione levels were independently increased by AFN at 6 and 24 h and further increased by LPS at 6 h compared to controls (Figure 4a,c). Oxidized glutathione was increased by LPS and AFN at 6 h and by LPS alone at 24 h compared to control (Figure 4b,d). At 24 h, AFN co-treatment attenuated the LPS-induced increase (Figure 4d). 

### 3.4. Decreases in Glutathione Do Not Affect IL-1β Expression

**Figure 5:**
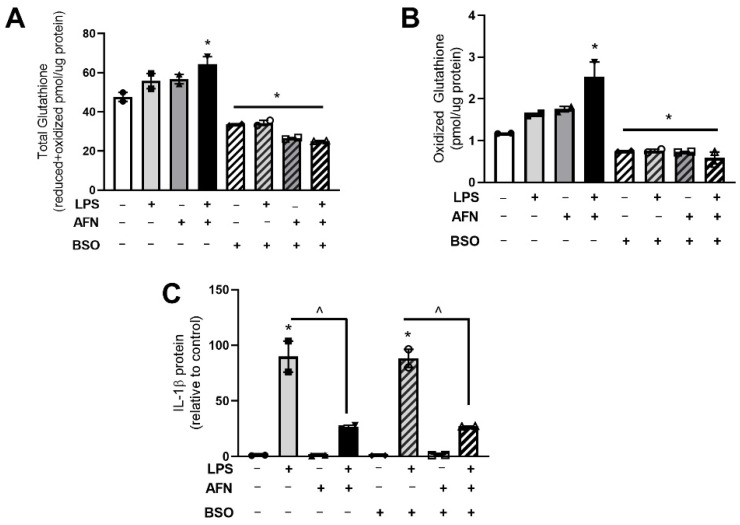
Total and oxidized glutathione are suppressed by BSO treatment with no effects on IL-1β levels. (**A**) Total glutathione (reduced + oxidized), (**B**) oxidized glutathione, and (**C**) IL-1β protein levels were measured in total cell homogenates at 6 h in LPS (0.05 mM)/BSO and AFN-treated MH-S cells as described in Methods. Solid bars indicate non-BSO treated controls, hatched bars indicate treatment with BSO. Data were analyzed by two-way ANOVA to identify the effects of LPS and/or AFN exposure. Tukey’s post-hoc analysis was used to determine significant differences between groups; *n* = 2 individual experiments. * *p* < 0.005 from control non-stimulated; ^ *p* < 0.005 LPS vs. LPS + AFN.

To evaluate the impact of LPS and AFN on de novo glutathione synthesis, MH-S cells were treated with LPS and/or AFN in the presence or absence of buthionine sulfoximine BSO. BSO inhibits the rate-limiting enzyme in glutathione synthesis (gamma-glutamylcysteine synthetase); thus, treatment with BSO prevented de novo glutathione synthesis. BSO decreased basal levels of total and oxidized glutathione (Figure 5a,b). The effects of BSO did not differ in cells cultured in the presence or absence of AFN or LPS. As previously demonstrated, AFN attenuated LPS-induced increases in Il-1β protein levels (Figure 5c) [12]. Il-1β protein levels did not differ between LPS + AFN-treated cells cultured in the presence or absence of BSO.

### 3.5. AFN-Mediated Attenuation of LPS-Induced IL-1β Expression Is Associated with NRF2 Binding to Il1β Promoter

**Figure 6:**
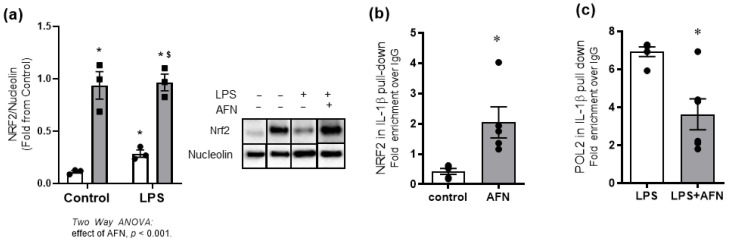
NRF2 nuclear localization and binding to IL1-β promoter. (**a**) Nuclear localization of NRF2 protein was identified at 1 h post AFN treatment. (**b**) Chromatin immunoprecipitation (ChIP) assay using IL-1β antibody revealed NRF2 protein directly binds to the *Il1b* promoter and (**c**) inhibits transcription by interfering with polymerase 2 (POL2) binding. White bars indicate DMSO control, and grey bars indicate AFN-treated. ChIP results were analyzed by Mann–Whitney, *n* = 3. For NRF2 nuclear localization, data were analyzed by two-way ANOVA to identify the effects of LPS and/or AFN exposure. Tukey’s post-hoc analysis was used to determine significant differences between groups; *n* = 3 individual experiments. * *p* < 0.005 from control non-stimulated. ^$^ *p* < 0.05 from control LPS.

Given previous evidence of NRF2 binding to the promoter of pro-inflammatory cytokine genes to directly suppress transcription, MH-S cells were cultured in the presence or absence of LPS and/or AFN [10]. Data revealed an independent effect of AFN but not LPS on NRF2 nuclear protein levels (Figure 6a). To evaluate the presence or absence of direct interactions between NRF2 and *Il1b* promoter, chromatin immunoprecipitation (ChIP) was performed. Our analyses identified direct binding of NRF2 to the *Il1b* promoter, and this interaction was significantly increased in AFN-treated cells (Figure 6b). To examine the relationship between enhanced NRF2 binding and attenuation of LPS-induced increases in *Il1b* expression, we evaluated the level of LPS-induced polymerase 2 (POL2) binding to the *Il1b* promoter. Our data revealed that LPS-induced POL2 binding was reduced by 20% in the presence of AFN (Figure 6c). 

## 4. Discussion

Gold-containing medications like AFN or ATG are FDA-approved for treatment of rheumatoid arthritis and have been used to treat inflammatory symptoms for decades [16]. Intriguingly, the mechanisms by which these compounds inhibit inflammation was largely unknown at the time they were adopted for clinical use. Subsequent investigation of gold compounds for alternative indications, including lung disease and cancer, have revealed the involvement of both NRF2 and NFκB pathways [17]. Thus, the NFκB pathway, as a key regulator of inflammation, represents an intersection between antioxidant and anti-inflammatory pathways modulated by NRF2. For a comprehensive review, the reader is directed to a recent publication by Saha and colleagues, which extensively summarizes key relationships between NRF2/ARE, inflammatory mediator expression, canonical and non-canonical NFκB pathway activation, and macrophage metabolism [18]. We have extensively studied the effects of AFN and ATG in the setting of hyperoxia exposure and have consistently observed decreases in inflammation, tissue injury, and cell death [2,5,6,19,20,21]. Despite our robust experimental evidence of decreased inflammation in lung injury models, the mechanisms by which gold compounds attenuate pro-inflammatory responses have not been revealed in our studies of lung epithelia. Given the significant contribution of macrophages toward lung inflammation and resolution, the present studies investigated the effects of gold compounds on lung macrophage inflammatory responses.

In the lung, Txnrd1 is most abundantly expressed in epithelia and macrophages, and AFN and ATG directly inhibit the activity of Txnrd1 [2]. Inhibition or deletion of Txnrd1 impairs antioxidant responses allowing increases in cellular ROS concentrations and activation of NRF2-dependent responses [5,22]. NRF2 is subsequently targeted to the nucleus and binds to cis elements within the DNA known as antioxidant response elements (AREs). AREs are found in many antioxidant genes and are responsible for upregulating cellular defenses (NRF2 pathway reviewed in [23]). Enhancement of the NRF2 activity has demonstrated protective effects in several models of lung injury [24,25,26,27,28]. As a result, modulation of NRF2 has become an attractive drug target for inflammatory diseases (reviewed in [27]).

Our data suggested that AFN attenuated the pro-inflammatory effects of LPS at the transcriptional level, as the LPS-induced increases in both *Il1β* mRNA and protein levels were markedly decreased by AFN treatment (Figure 2) [4,6,12]. Cross-talk between NRF2 and NFkB pathways has been extensively characterized (reviewed in [29]). Although LPS clearly increased NFκB pathway activation, AFN treatment had no effect on critical signaling events leading to canonical NFκB activation, including phosphorylation of IκBα or p65 NFκB, or degradation of IκBβ (Figure 3). We also evaluated the expression of the NFκB-regulated transcript *Nlrp3*, a key regulator of IL-1β-dependent responses. Though LPS increased *Nlrp3* expression, AFN had no effect, suggesting that anti-inflammatory effects did not involve modulation of the NLRP3 inflammasome. Others have demonstrated that increases in NRF2 expression prevents the assembly of NLRP3, but this mechanism was not investigated in our studies [11,30]. Collectively, our findings suggested that AFN-mediated decreases in IL-1β expression are likely at the transcriptional level and are NFκB-independent. AFN treatment increases NRF2 nuclear localization leading to increased expression of antioxidant genes, specifically those involved in de novo glutathione synthesis [5]. Glutathione has potent anti-inflammatory and antioxidant effects and, we speculated, could contribute to decreases in IL-1β expression. AFN treatment of MH-S cells resulted in increased total glutathione levels at both 6 and 24 h, which is similar to our findings in lung epithelia (Figure 4) [2]. In addition, GSSG levels in AFN-treated cells were lower than controls at the 24 h time point. It is plausible that these changes may confer a reducing intracellular environment, thus decreasing inflammation. To address AFN-mediated increases in GSH-dependent antioxidant defenses as the mechanism for decreases in IL-1β expression, we inhibited GSH synthesis (Figure 5). While LPS + AFN treatment increased total GSH levels, BSO co-treatment had no effect on LPS + AFN-induced decreases in IL-1β expression. These data suggest that AFN-mediated decreases in IL-1β in lung macrophages are not mediated by enhanced GSH synthesis.

Previous reports in bone marrow-derived macrophages evaluated the effects of AFN-mediated NRF2 activation on IL-1β transcription. This report utilized chromatin immunoprecipitation and identified the presence of NRF2 in the *Il1b* promoter region (Figure 6 and Supplementary Material Figure 6) [12]. Our analysis revealed similar findings: NRF2 immunoprecipitated with the *Il1b* promoter, an effect that was significantly increased in the presence of AFN. Our findings of inverse effects of AFN on POL2 binding within the immunoprecipitation complex provides further evidence that AFN attenuates LPS-induced increases in IL-1β expression via NRF2-dependent transcriptional repression.

Our data in lung macrophages demonstrated that AFN-induced NRF2 activation directly suppresses IL-1β synthesis independent of NFκB and glutathione-mediated antioxidant mechanisms. These findings are in contrast to our data in lung epithelia in which protective effects of AFN were glutathione dependent. Though our characterization of lung macrophages was performed in a transformed cell line, which may respond somewhat differently than primary cells, our findings are in alignment with similar effects of NRF2 induction in bone marrow-derived primary macrophages [12]. Our data also differ from those by Isakov et al., who reported that AFN attenuated LPS-induced increases in NLRP3 [4]. In contrast to our studies, those performed by Isakov et al. were conducted in immortalized peritoneal macrophages. Collectively, the differences in our data and those generated by other groups highlight the challenges in extrapolating findings between similar cell types derived from different sources and suggest the need to confirm findings in specific cell types.

## 5. Conclusions

We and others continue to explore the utility of NRF2 activators to mitigate acute lung injury. Importantly, NRF2 single nucleotide polymorphisms have been linked to ARDS susceptibility in humans [31,32]. Thus, NRF2 activation remains a compelling therapeutic target to improve outcomes in patients with pulmonary diseases, as extensively reviewed by Liu and colleagues [23]. Indeed, the pleiotropic effects of NRF2 activators in the context of lung development, injury, and repair continue to be elucidated. The findings in the present study further define mechanisms by which potent NRF2 activation attenuates pulmonary inflammation. In summary, our data indicate that AFN-mediated IL-1b reduction occurs via cis binding of NRF2 for transcriptional suppression and does not affect LPS-induced activation of NF-kB signaling pathways. Collectively, our research suggests that gold compounds elicit NRF2-dependent pulmonary protection by suppressing macrophage-mediated inflammation and by enhancing glutathione-dependent epithelial antioxidant responses.

## Data Availability

Data is contained within the article.

## Supplementary Materials

The following are available online at https://www.mdpi.com/article/10.3390/antiox10050632/s1.
