# Supplementary material for: Auranofin-Mediated NRF2 Induction Attenuates Interleukin 1 Beta Expression in Alveolar Macrophages
Source: Antioxidants (Basel). 2021 Apr 21;10(5):632. doi: 10.3390/antiox10050632 (PMC8143169; doi:10.3390/antiox10050632)
Supplement: Supplementary file 1 [file antioxidants-10-00632-s001.zip › antioxidants-1138724-supplementary.pdf]

Figure 3 a

031417, Total IκBα IB

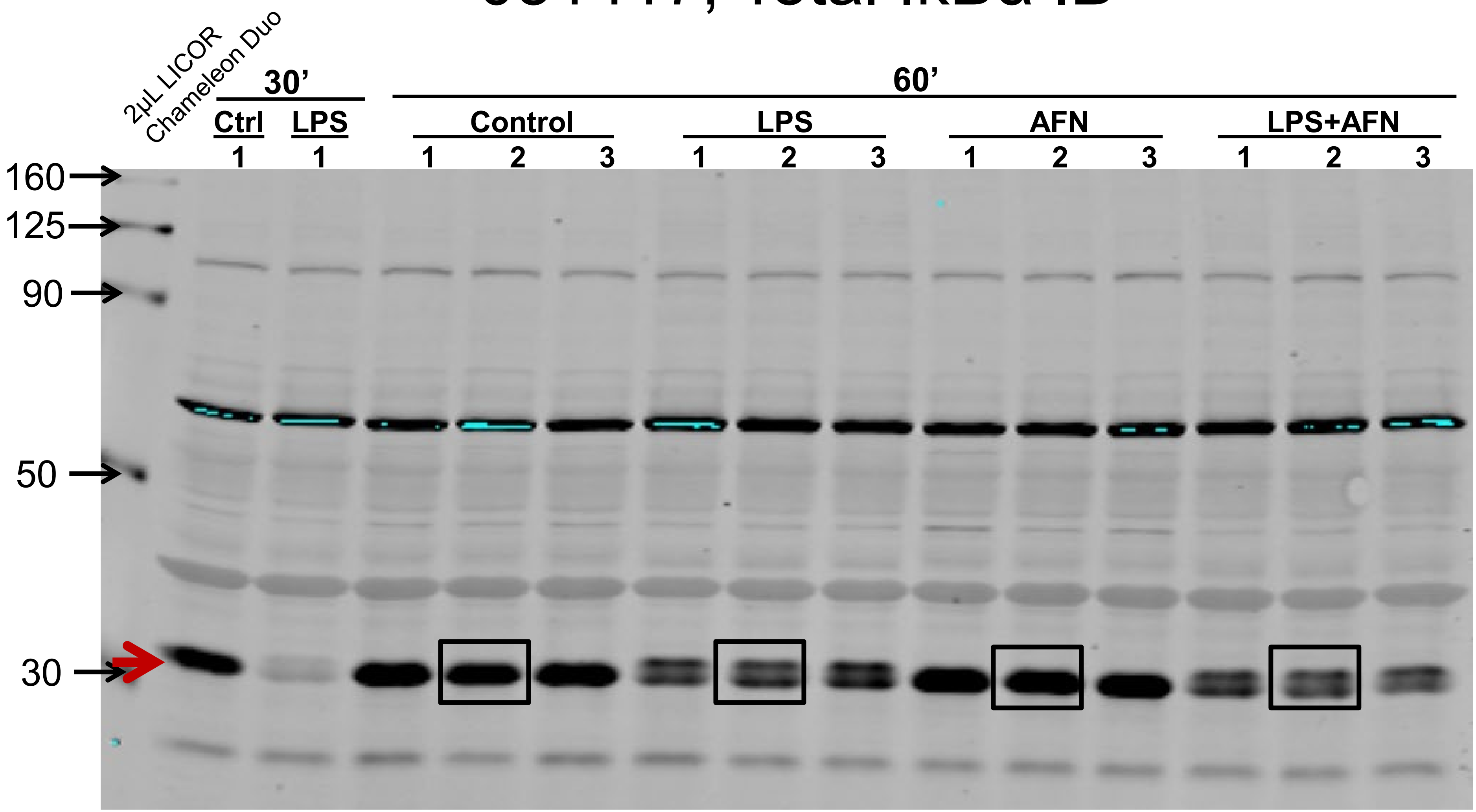

10% SDS PAGE
50µg per lane
1hr 100V, 4deg Transfer to Nitrocellulose Membrane
1hr block, rm temp, in 5% Milk-TBST
O/N, 4 deg, IκBα (1:1,000) in 5% Milk-TBST
1hr, rm temp, Anti-Mouse 680RD (1:20,000) in 5% Milk-TBST

Expected Size: 40 kDa

Figure 3 b

030817, P-NFκB-p65 IB

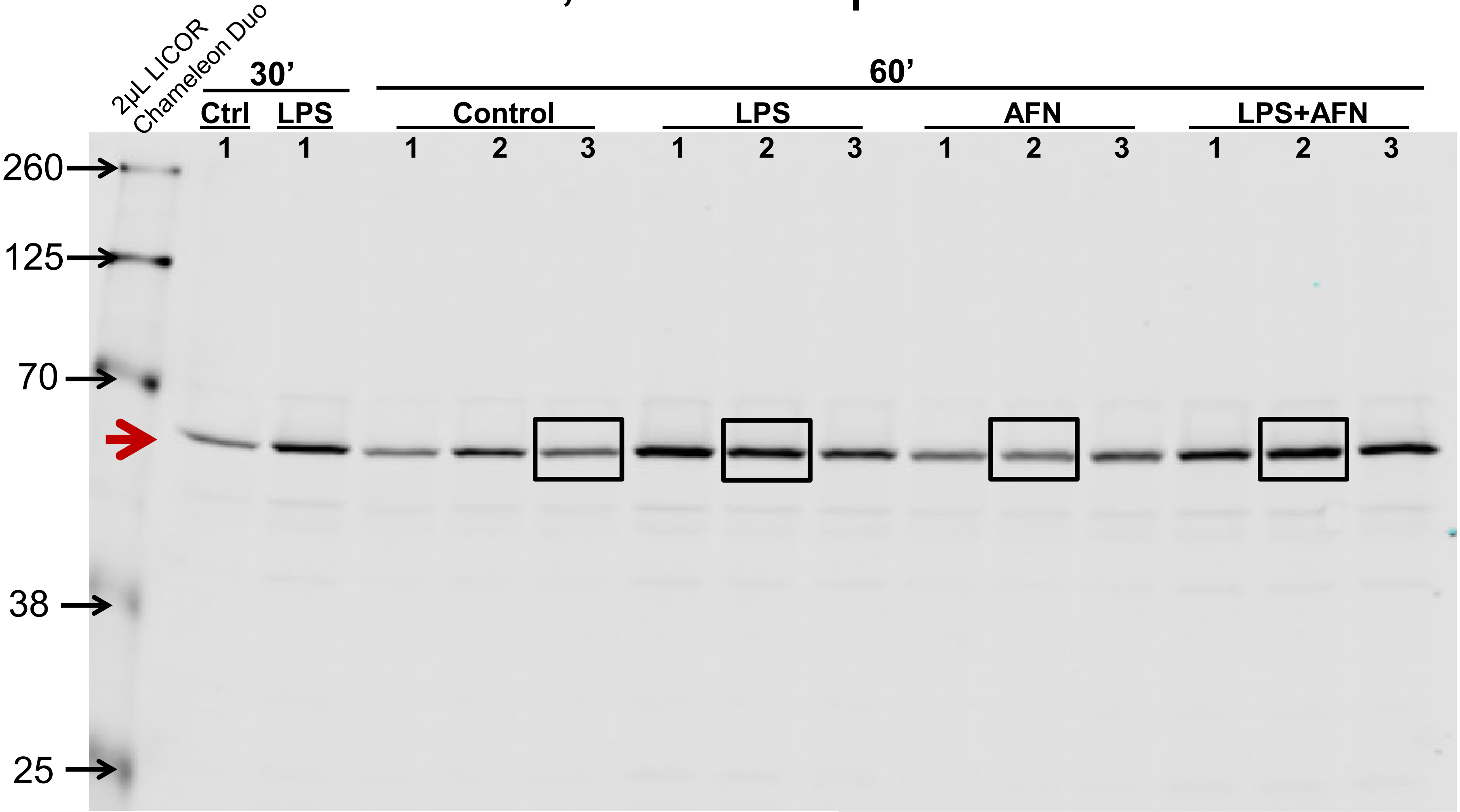

10% SDS PAGE
50µg per lane
1hr 100V, 4deg Transfer to Nitrocellulose Membrane
1hr block, rm temp, in 5% Milk-TBST
O/N, 4 deg, P-NFκB-p65 (1:1,000) in 5% BSA-TBST
1hr, rm temp, Anti-Rabbit 800CW (1:20,000) in 5% Milk-TBST

Expected Size: 65 kDa

Figure 3 c

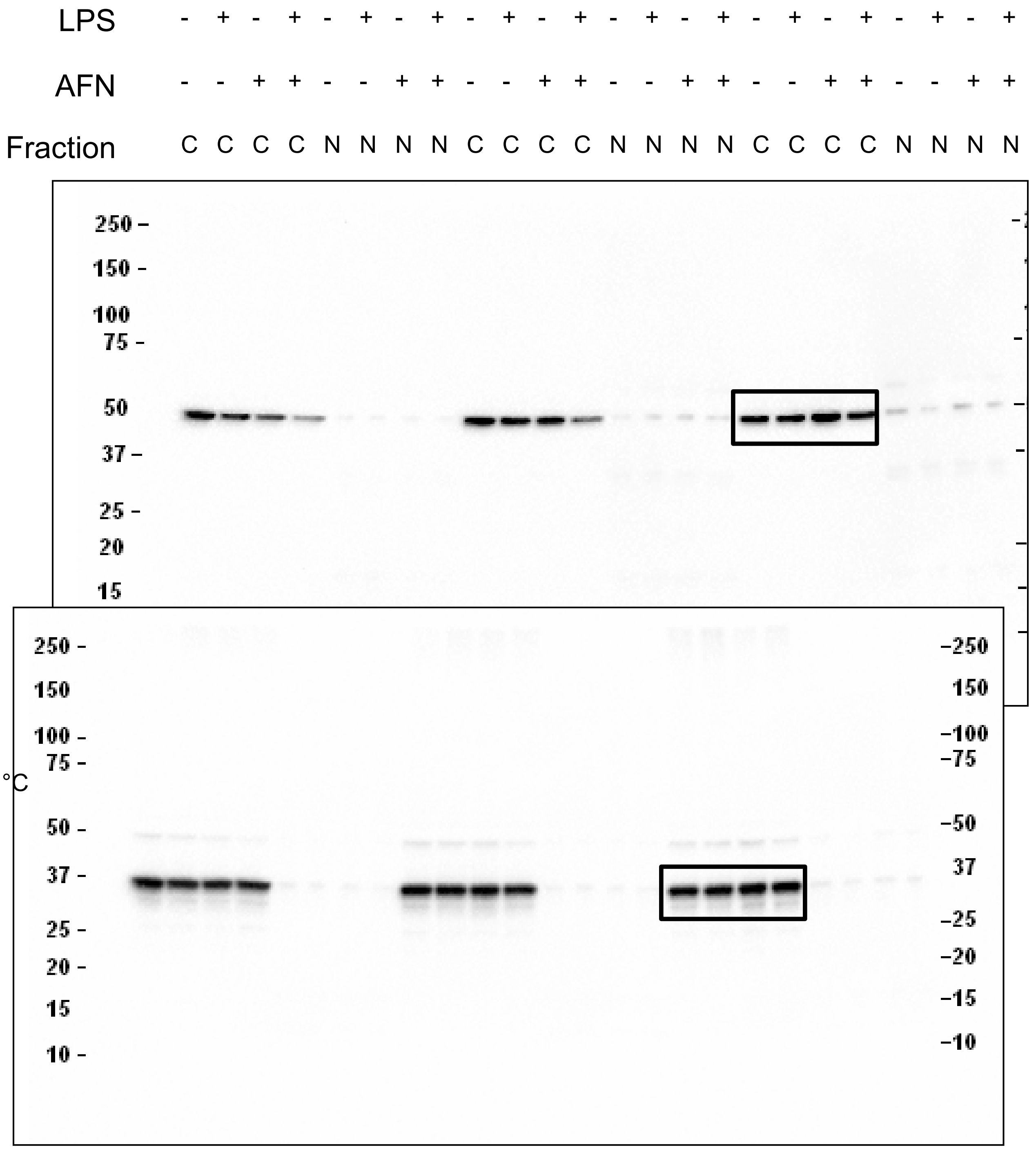

IKB-beta blot:
1:1000 IKB-b in 5% milk TBST O/N in 4 °C
1:5000 mouse IgG-HRP from R&D
Exposure 17 seconds

IKB-beta:
Santa Cruz sc-74451
IκB-β (D-3)

Expected size: 45kDa

GAPDH blot:
1:4000 GAPDH in 5% BSA TBST O/N in 4 °C
1:5000 mouse IgG-HRP from R&D
Exposure 1.1 seconds

GAPDH details:
Millipore MsxGAPDH MAB374
Mouse monoclonal
Lot# 2430317

Expected size: 36kDa

Figure 6 a - Nrf2

1 hour treated samples only (samples are paired with respective non-nuclear (C) and nuclear (N)):

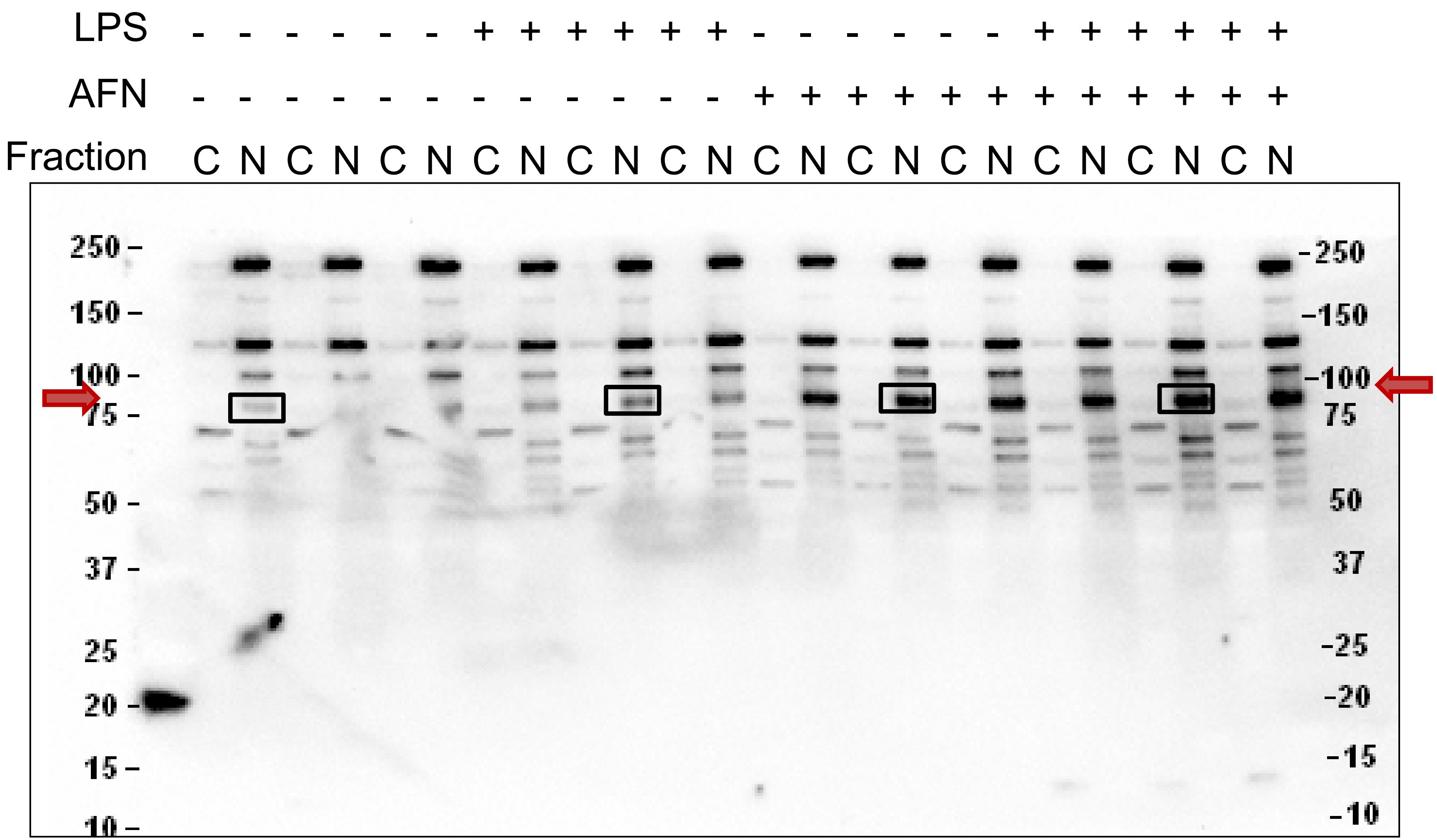

Blocked 5% milk TBST 1hr
1:500 Nrf2 overnight
1:5000 anti-Rabbit HRP 1 hr
Exposure was ~25sec

Nrf2 Expected Size: 80 kDa

Figure 6 a - Nucleolin

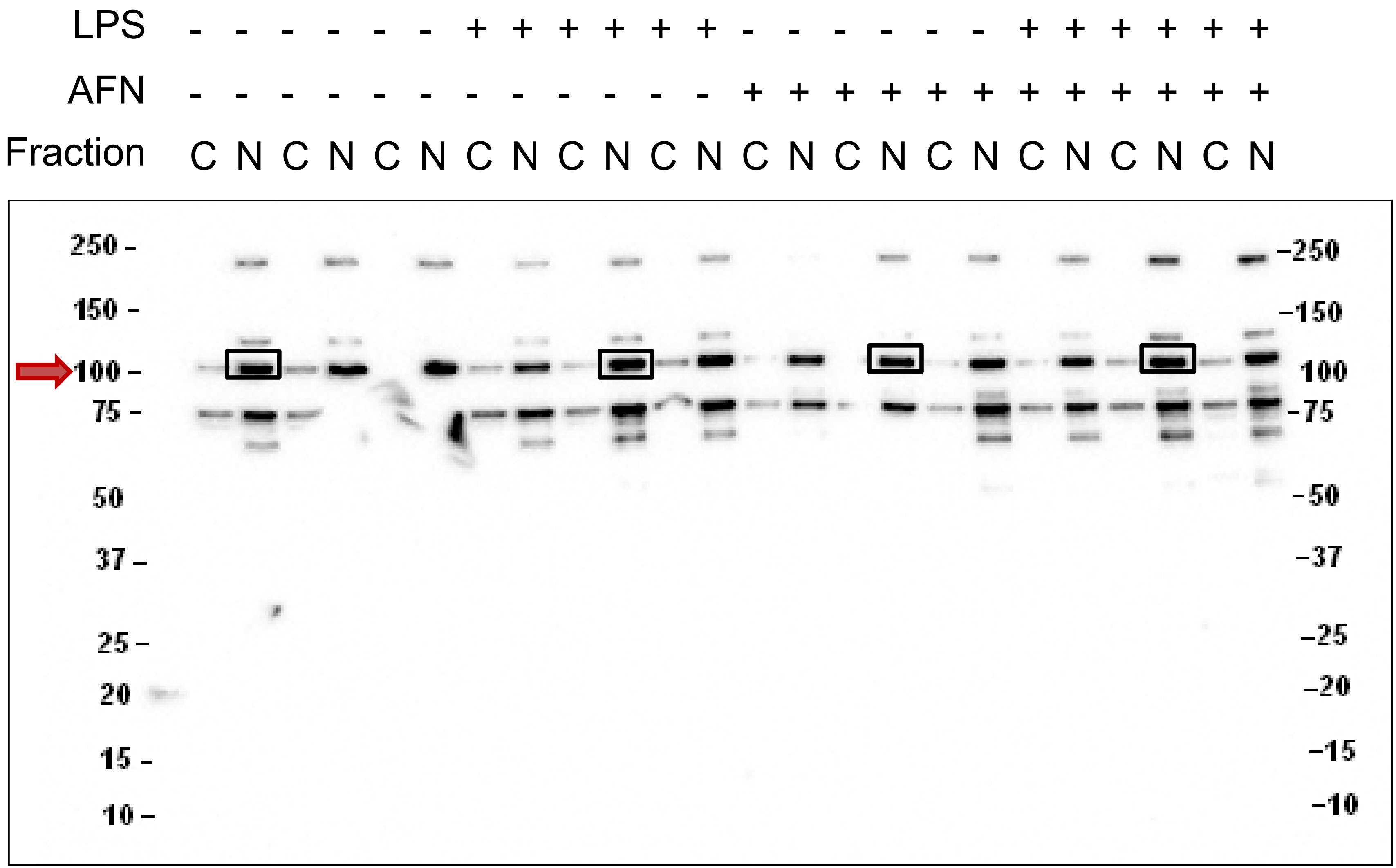

Anti-Nucleolin ab22758
1:5000 5% milk TBST 2hr
Anti-Rabbit-HRP 1:5000 in
TBST 1 hr
Exposure time was 0.27 sec

Expected size: 100 kDa
